# Probiotics are a good choice for the treatment of bacterial vaginosis: a meta-analysis of randomized controlled trial

**DOI:** 10.1186/s12978-022-01449-z

**Published:** 2022-06-13

**Authors:** Rongdan Chen, Rong Li, Wei Qing, Yingxuan Zhang, Zuyi Zhou, Yi Hou, Yiya Shi, Hongwei Zhou, Muxuan Chen

**Affiliations:** 1 Department of Laboratory Medicine, ZhuJiang Hospital, Southern Medical University, Guangzhou, Guangdong China; 2 Microbiome Medicine Center, Zhujiang Hospital, Southern Medical University, Guangzhou, Guangdong China

**Keywords:** Probiotics, Bacterial vaginosis, Cure rate, Meta-analysis

## Abstract

**Background:**

Bacterial vaginosis (BV) is one of the most common vaginal infectious diseases in female reproductive period. Although the existing view is that probiotic treatment may be one of the feasible methods for the treatment of BV, different intervention methods lead to different treatment results. Therefore, up-to-date and comprehensive evidence in this regard is essential for the development of intervention strategies.

**Objective:**

This meta-analysis aims to systematically evaluate the role of probiotics in the treatment of BV in adult women.

**Methods:**

We searched the databases of Embase, Cochrane Library, PubMed, Web of Science and ClinicalTrials.gov for Randomized Controlled Trials published until November 7, 2021. Meta-analysis was performed by Revman5.3 software to systematically evaluate the clinical efficacy of probiotics adjunctive therapy in the treatment of BV. The literatures were screened and evaluated according to the inclusion and exclusion criteria. Chi-square test was used to test the heterogeneity between trials. Random or Fixed effect models were used to analyze the cure rate of BV.

**Results:**

Fourteen randomized controlled trials compared the efficacy of probiotics with antibiotic therapy (probiotics + antibiotics group) versus antibiotics alone or plus placebo (antibiotics (+ placebo) group) for BV [Risk Ratios (RR) = 1.23, 95% CI (1.05, 1.43), P = 0.009]. Three compared the efficacy of probiotics regimen (probiotics group) and antibiotics (antibiotics group) in the treatment of BV [RR = 1.12, 95% CI (0.60, 2.07), P = 0.72]. Another Three compared the efficacy of probiotics regimen (probiotics group) with placebo (placebo group) [RR = 15.20, 95% CI (3.87, 59.64), P < 0.0001].

**Conclusion:**

Our meta-analysis suggests probiotics may play a positive role in the treatment of BV, but more strong evidence is needed.

## Plain Language Summary

Our meta-analysis found that probiotics may play an active role in adjuvant treatment of bacterial vaginosis by conventional antibiotic therapy. It was emphasized that oral administration of *L. rhamnose* was more effective than vaginal application of *L. rhamnose* in the treatment of bacterial vaginosis. The therapeutic effect of probiotics varies with the administration route and dosage of probiotics.

## Introduction

As one of the most common vaginal infectious diseases in the child-bearing period of women [1], bacterial vaginosis is caused by the imbalance of microecology in the vagina and the mixed infection of *Gardnerella Vaginalis* (GV) and anaerobic bacteria owning to regular irrigation, multiple sexual partners, non-condom use, smoking and reduced estrogen levels [1–4]. BV patients often have symptoms including increased secretion of vaginal discharge, fishy smell in leucorrhea, and pruritus and burning in vulvas. In addition, some studies have shown that BV is likely to cause a range of health problems such as premature birth, pelvic inflammation, infection and transmission of sexually transmitted diseases including acquired immune deficiency syndrome [5–11]. And because of the obvious discomfort of vulva when BV onsets and the high recurrence rate of BV, women's life quality and even their mental health are significantly negatively affected by BV, although the prevalence of BV varies geographically [5].

Approximately 50% of BV patients have clinical symptoms, which can be diagnosed by Amsel standard or Nugent score. Among them, Amsel standard is a convenient and practical method, which is widely used as the gold standard in clinic [1, 12]. If possible, the vaginal flora could also be graded and evaluated by Nugent scoring system [13, 14]. Nugent scoring system diagnosis of BV shows a higher sensitivity and lesser dependence on clinicians.

Since the vaginal microbiota of BV patients has changed from *Lactobacillus*, the dominant microbiota of vagina, to a more diversified community mainly composed of facultative and obligate anaerobic bacteria. Nowadays, antibiotics such as metronidazole and clindamycin are used worldwide in clinical treatment to fight against BV related microbes in a short period to give space for normal vaginal microbiota to restore [15, 16]. However, an extremely high recurrence rate of 69% could be observed in patients after effective antibiotic treatment. And there may be some adverse effects like gastrointestinal discomfort such as nausea and vomiting from antibiotic use, as well as the risk of developing resistance to antibiotics [3, 9, 17]. Therefore, it is crucial to explore a safer and more long-lasting clinical treatment for BV. Fortunately, probiotics preparations have been proved to be a safe alternative for restoring the microecological balance of female reproductive tract and they are generally accepted by patients [18]. However, even though more and more Randomized Controlled Trials (RCTs) of using probiotics as an alternative or adjunctive treatment for BV have been reported, they result in a controversial efficacy. There are also obvious differences in dosage regimens of probiotics in previous studies [19–23].

This study aimed to clarify the efficacy and role of probiotics in BV treatment by adopting meta-analysis to integrate scattered literatures and systematic analysis to explore the source of heterogeneity and its impact on trial results.

## Methods

### Search strategy

Literature retrieval was conducted independently by two researchers. The Cochrane Library, PubMed, EMBASE, Web of Science databases and ClinicalTrials.gov website were searched for RCTs on probiotics in the treatment of bacterial vaginosis that were published prior to 7 November 2021. We searched the literature using subject terms and free words, including terms related to or included “Vaginosis, Bacterial”, including Bacterial Vaginitides; Vaginitides, Bacterial; Bacterial Vaginosis; Vaginitis, Nonspecific; Nonspecific Vaginitis; Bacterial Vaginoses; Vaginoses, Bacterial; Bacterial Vaginitis; Vaginitis, Bacterial. Words related to “Probiotics” or "Lactobacillales", including Lactic Acid Bacteria, Lactobacillus, Lactobacilli, Bifidobacterium, and LB were also searched. The study protocol was registered on PROSPERO (CRD42021289871).

### Inclusion and exclusion criteria

The following were the inclusion criteria for considering full-text publications: (a) studies must be RCTs; (b) study population was women in childbearing age who were non-pregnant and were diagnosed only with BV by either Nugent score [13] or Amsel criteria [1]; (c)intervention for experimental group was probiotics only (regardless of dose, route of administration, single or mixed strain) or probiotics in combination of conventional antibiotics treatment matched with antibiotics or placebo as control; (d) the prioritized treatment outcomes was cure or recurrent rate of BV.

The exclusion criteria were articles that (a) studies which included pregnant women, women with sexually transmitted infections or other urinary tract infections other than BV; (b) had no full text available or was not written in English; (c) failed to report the required results; (d) had unextractable outcome indicators. For example, those studies which barely demonstrated the cure or recurrence rate without the detailed number of cured or recurrent participants.

### Determination of main outcome indicators

The main outcome indicator was higher BV cure rate in the probiotics group against placebo group or the antibiotic group, which was evaluated by cure corresponds to the diagnostic criteria. In some articles, the outcome index was the percentage of the recurrence rate, which we had converted to the cure rate for evaluation. Cure refers to the normalization of diagnostic indicators, such as Amsel criteria ≤ 1 or Nugent score ≤ 3.

Secondary observation indicators included (a) disappeared clue cells, negative in sialidase test, and had no symptoms and signs of BV (such as no unpleasant secretion or odor); (b) normal vaginal flora; (c) prolonged time of recurrence after initial treatment when adjuvant therapy with lactic acid bacteria was used; (d) improved Nugent score to below 7 after treatment.

The most common local adverse events were abnormal vaginal discharge, abnormal vaginal odor, external genital irritation and genital pruritus. Safety was assessed by recording all side effects. Adverse events that occurred during the trial were evaluated in the treatment group and the placebo group to determine whether there was a significant difference between the two groups.

### Data extraction and synthesis

Data were extracted in tables, including author, year of publication, type of study, age, sample size (intervention/control), intervention measures (type, dosage, drug-delivery way, intervention time), follow-up time, and diagnosis criteria.

### Quality assessment of the studies

Cochrane Handbook for Systematic Reviews of Interventions for assessing risk of bias was recommended quality assessment method used in randomized controlled trials, which mainly includes 7 aspects. Two researchers (Chen and Li) conducted data extraction and risk assessment respectively. Any differences were discussed and a third investigator (Qing) was presented to decide whether to reach an agreement.

### Statistical analysis

RevMan5.3 software was used for statistical analysis of the included data. The statistical method was expressed as Mantel–Haenszel (M-H), and the effect measure as RR and 95% Confidence Interval (CI). P < 0.05 indicated that the difference was statistically significant. Chi-square test was used for heterogeneity analysis. If P > 0.1 and I^2^ ≤ 50%, the heterogeneity between studies was low. If P ≤ 0.1 and I^2^ > 50%, it indicated that there was significant heterogeneity between studies. Due to the different clinical designs of these randomized controlled trials, fixed effect models were used for meta-analysis of trials with low heterogeneity. For trials with high heterogeneity, we used random effect models for meta-analysis, and subgroup analysis or impact analysis.

Subgroup analysis was to find out the causes of heterogeneity by grouping the route of administration, diagnostic criteria, recruitment area, follow-up time, species of probiotics, use of *L. rhamnose* and dosage of probiotics. Sensitivity analysis was to gradually exclude the included literature and recalculate the I^2^ and P values. If heterogeneity has changed greatly after the exclusion of an article, it may be the main source of heterogeneity.

## Results

### Study identification and selection

**Fig. 1:**
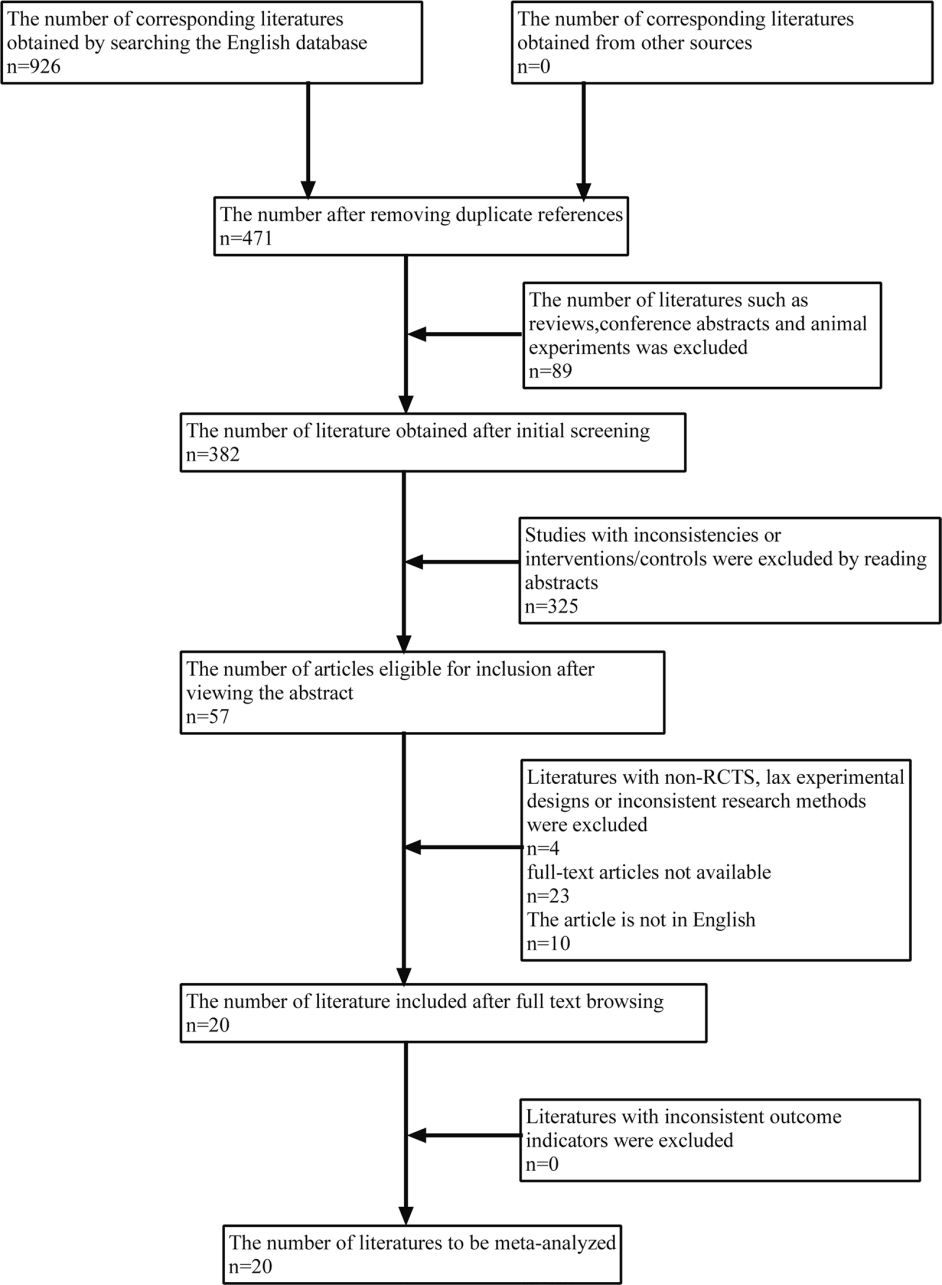
PRISMA flow diagram

According to the established retrieval strategy, 926 relevant literatures were preliminarily retrieved. After removing the repeated 455 literatures, there were 471 literatures that can be screened. After excluding reviews, meeting minutes, and others non relevant article types, 382 papers remained. After reading the title and abstract, there were 57 articles left. An additional 37 articles were excluded based on inclusion and exclusion criteria. Finally, 20 relevant articles were included in this study, involving 2093 participants. The flowchart shows the process of literature selection (Fig. 1).

### Risk of bias assessment

**Fig. 2:**
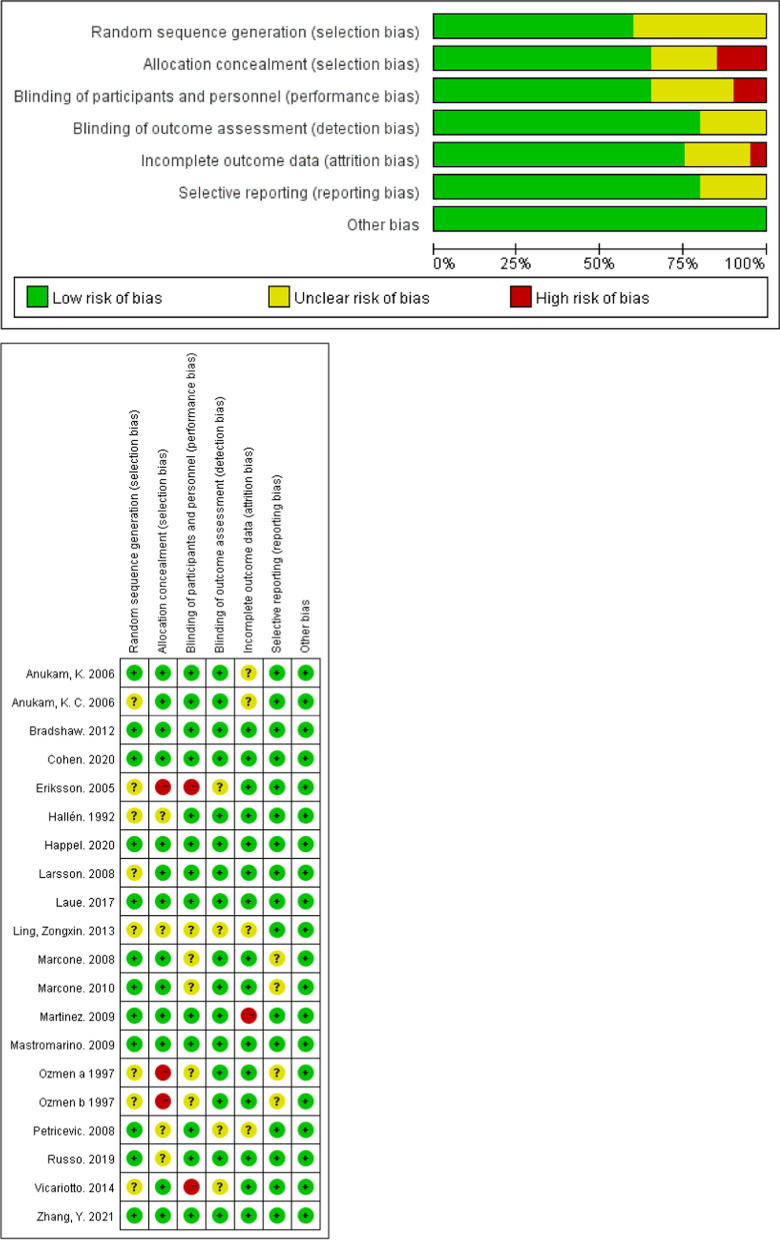
Risk of bias assessment

The assessment of risk of bias for the included 20 RCTs are shown in Fig. 2. Risk of bias were mainly derived from Random Sequence Generation, eight studies in this section had uncertain bias risk. Six of the included studies achieved a score of seven, indicating good quality. Overall, the quality of the included studies was moderate. Of the 20 studies, most had an uncertain risk of bias, and only five were considered high risk of bias.

### Characteristics of the studies

The main characteristics of the 20 randomized controlled trials included in this meta-analysis are shown in Table 1. The included trials were published between 1992 and 2021, and consist of 1067 patients in the experimental group and 1026 patients in the control group. These twenty articles can be divided into three cases according to different experimental schemes. Fourteen randomized controlled trials compared the efficacy of antibiotics in addition to probiotics in BV (antibiotics + probiotics group) and antibiotics alone (or with placebo) in BV (antibiotics (+ placebo) group). Three trials compared the efficacy of probiotics (probiotics group) with antibiotics (antibiotics group) for BV, and three randomized controlled trials compared the efficacy of probiotics (probiotics group) with placebo (placebo group) for BV.

**Table 1:** Characteristics of the included studies in the meta-analysis

Reference	Type of study	Age (Mean ± SD)	Sample size (Intervention/Control)	Intervention				Control				Follow-up time	Diagnosis criteria
				Probiotic type	Dosage	Drug-delivery way	Time	Antibiotic type	Dosage	Drug-delivery way	Time		
Anukam et al., 2006	R, DB, PC	18–44	106 (49/57)	*L. rhamnosus* gR-1, *L. reuteri* RC-14	10^9^ CFU × 2/d	Take orally	30d	Metronidazole	500 mg	Take orally	7 days	30 d	Nugent score 7–10
				Metronidazole	500 mg × 2/d	Take orally	7 d						
Bradshaw et al., 2012	R, DB, PC	18–50	268 (133/135)	*L. acidophilus* KS400	> 10^7^ CFU	Vaginally	12 d	Placebo	/	Vaginally	12 days	3 m	Nugent Score 7–10
				metronidazole	400 mg × 2/d	Take orally	7 d	Metronidazole	400 mg	Take orally	7 days		
Cohen et al., 2020	R, DB, PC	30.7 ± 6.8/ 31.4 ± 7.1	228 (133/64)	*L. crispatus* CTV-05	2 × 10^9^ CFU	Vaginally	30 d	Placebo	/	Vaginally	30 days	12 w	Amsel’s criteria ≥ 3
				metronidazole	0.75%	Vaginally	5 d	Metronidazole	0.75%	Vaginally	5 days		
Eriksson et al., 2005	R, DB, PC	32 (20–52)/ 32 (18–53)	197 (91/96)	*L. gasseri,* *L. casei rhamnosus,* *L. fermentum*	10^8^ CFU	Vaginally	> 5 d	Placebo		Vaginally	> 5 days	28 d	Amsel’s criteria ≥ 3
				Clindamycin	100 mg	Vaginally	3 d	Clindamycin	100 mg	Vaginally	3 days		
Happel et al., 2020	R, OB	18–45	29 (18/11)	*L. acidophilus*, *L. rhamnosus* GG,	≥ 2 × 10^9^ CFU	Take orally&Vaginally	15d	Metronidazole	0.75%	Vaginally	5 days	5 m	Nugent score 7–10
				metronidazole	0.75%	Vaginally	5 d						
Larsson et al., 2008	R, DB, PC	≥ 18	100 (50/50)	*L. gasseri* *L. rhamnosus*	≥ 10^8–9^ CFU	Vaginally	10d	Placebo	/	Vaginally	10 days	30 d	Amsel’s criteria ≥ 3
				Clindamycin	2%	Vaginally	7 d	Clindamycin	2%	Vaginally	7 days		
Laue et al., 2017	R, DB, PC	32.6 ± 11.2/ 39.0 ± 12.3	34 (17/17)	*L. crispatus,* *L. gasseri,* *L. rhamnosus,* *L. jensenii*	10^7^ CFU/ml × 2/d	Orally	4 w	Placebo	/	Orally	4 weeks	28 d	Amsel’s criteria ≥ 3
				Metronidazole	500 mg × 2/d	Orally	7 d	Metronidazole	500 mg × 2/d	Orally	7 days		
Marcone et al., 2008	R, NB	18–40	84 (42/42)	*L. rhamnosus*	> 40,000 CFU	Vaginally	Once a week /2 m	Metronidazole	500 mg × 2/d	Orally	7 days	180 d	Amsel’s criteria = 4
				Metronidazole	500 mg × 2/d	Orally	7 d						
Marcone et al., 2010	R, DB	18–45	46 (23/23)	*L. rhamnosus*	40,000 CFU	Vaginally	Once a week /6 m	Metronidazole	500 mg × 2/d	Orally	7 days	30 d	Amsel’s criteria = 4
				Metronidazole	500 mg × 2/d	Orally	7 d						
Martinez et al.,2009	R, DB, PC	30.0 ± 10.9/ 30.3 ± 10.7	64 (32/32)	*L. rhamnosus* GR-1, *L. reuteri* RC-14	1 × 10^9^ CFU	Orally	28 d	Placebo	/	Orally	28 d	28 d	Amsel’s criteria ≥ 3 & Nugent score 7–10
				Tinidazole	2 g	Orally	28 d	Tinidazole	2 g	Orally	28 d		
Ozmen et al., 1997b	R, NB	18–53	210 (96/114)	*L. acidophilus*	10^7^–7 × 10^8^ CFU	Vaginally	12d	Metronidazole	500 mg × 2/d	Orally	7 days	22–35 d	Amsel’s criteria ≥ 3
				Metronidazole	500 mg × 2/d	Orally	7 d						
Petricevic et al., 2008	R, OB	18–45	171 (83/88)	*L. casei rhamnosus*	10^9^ CFU	Vaginally	7d	Clindamycin	300 mg × 2/d	Orally	7 days	28 d	Nugent score 7–10
				Clindamycin	300 mg × 2/d	Orally	7 d						
Russo et al., 2019	R, DB, PC	18–50	48 (24/24)	*L. acidophilus* GLA-14, *L. rhamnosus* HN001	5 × 10^9^ CFU	Orally	15 d	Placebo	/	Orally	15 days	6 m	Nugent score > 7
				Metronidazole	500 mg × 2/d	Orally	7 d	Metronidazole	500 mg × 2/d	Orally	7 days		
Zhang et al., 2021	R, NB	18–65	99 (52/47)	*L. rhamnosus* GR-1 *L. reuteri* RC-14	≥ 1 × 10^9^ CFU	Orally	30 d	Metronidazole	0.2 g	Vaginally	7 days	90 d	Nugent score ≥ 7
				metronidazole	0.2 g	Vaginally	7d						
Anukam, K. C et al.,2006	R	18–50	35 (17/18)	*L. rhamnosus* GR-1, *L. reuteri* RC-14	10^9^ CFU	Vaginally	5 d	Metronidazole	0.75%	Vaginally	5 days	30 d	Nugent score 7–10
Ling, Zongxin et al., 2013	R	/	55 (25/30)	*L. delbrueckii(*lactis DM8909)	≥ 10^9^ CFU	Vaginally	10 d	Metronidazole	500 mg	Vaginally	7 days	30d	Amsel’s criteria ≥ 3 &Nugent score7–10
Ozmen et al., 1997a	R, NB	18–53	211 (97/114)	*L. acidophilus*	10^7^–7 × 10^8^ CFU	Vaginally	12 d	Metronidazole	500 mg × 2/d	Orally	7 days	22-35d	Amsel’s criteria ≥ 3
Hallén et al.,1992	R, DB, PC	17–40	57 (28/29)	*L. acidophilus*	10^8–9^ CFU	Vaginally	6 d	Placebo	/	Vaginally	6 days	20d	Amsel’s criteria ≥ 3
Mastromarino et al., 2009	R, DB, PC	33 ± 9.9/ 35 ± 9.2	34 (18/16)	*L. brevis* CD2, *L. salivarius* FV2, *L. plantarum* FV9	10^9^ CFU	Vaginally	7 d	Placebo	/	Vaginally	7 days	28d	Amsel’s criteria ≥ 3
Vicariotto et al., 2014	R, DB, PC	34.7 ± 8.9	34 (24/10)	*L. fermentum* LF15 *L. plantarum* LP01	4 × 10^9^ CFU	Vaginally	7 d	Placebo	/	Vaginally	7 days	28d	Amsel’s criteria ≥ 3 &Nugent score7–10

R: randomized; DB: double blind; PC: placebo controlled; NB: not blind; OB: observer blind; CFU: colony-forming units

### Meta-analysis of treatment efficacy

**Fig. 3:**
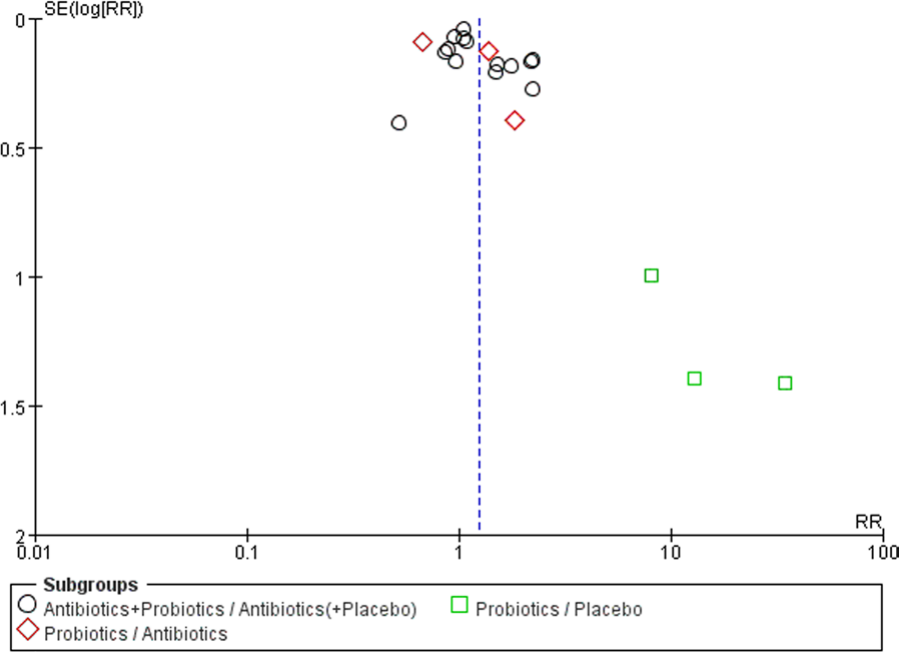
Funnel plot for risk of bias

A total of 20 RCTs were included in this study which were divided into three groups (G1, G2, G3) for analysis according to the different intervention methods. Funnel plots suggested the heterogeneity between those studies (Fig. 3).

**Fig. 4:**
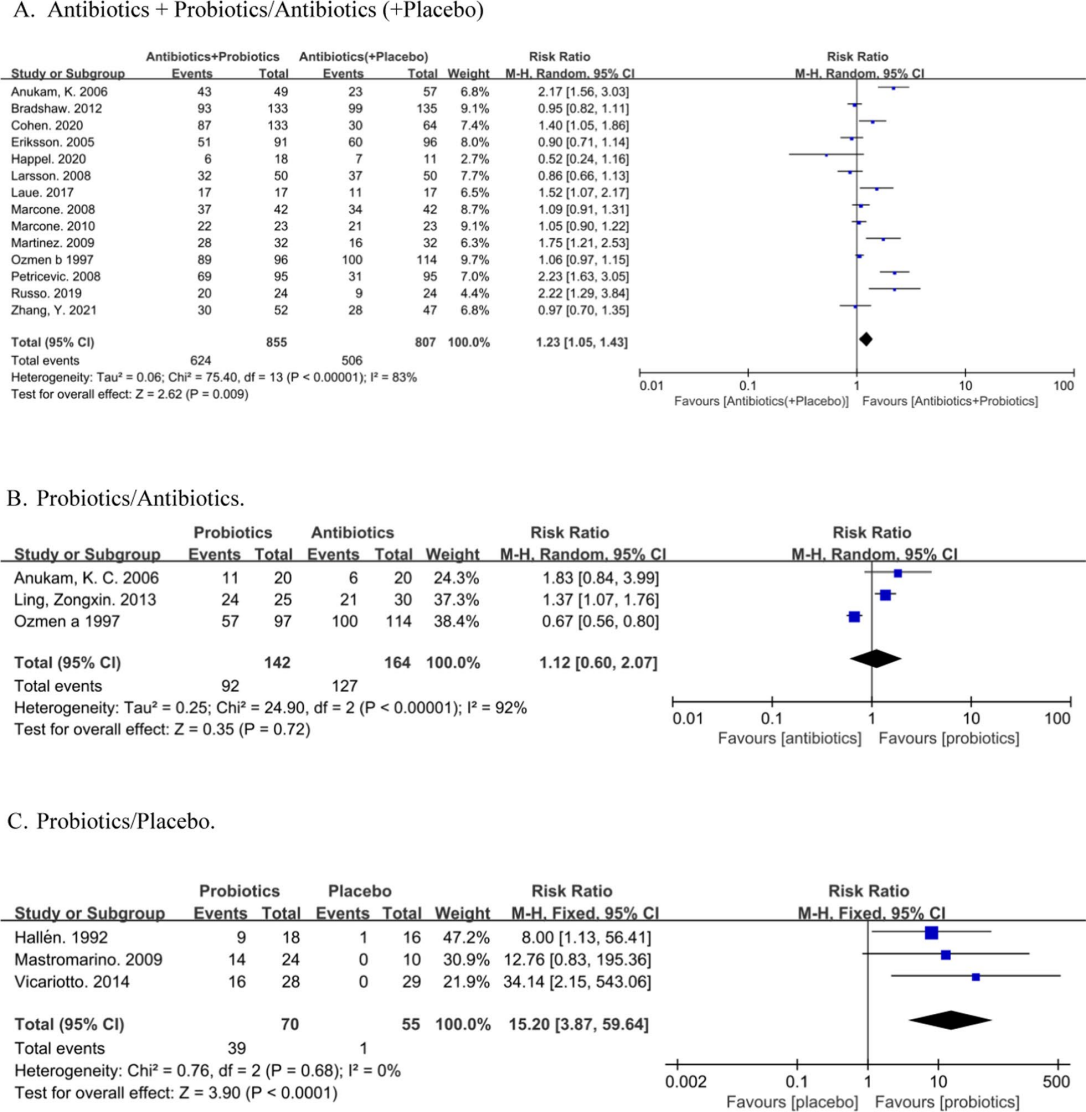
Forest plot of efficacy outcome. **A** Forest plot of Antibiotics + Probiotics/Antibiotics (+Placebo), used the random effect model. **B** Forest plot of Probiotics/Antibiotics, used the random effect model. **C** Forest plot of Probiotics/Placebo, used the fixed effect model

G1: Fourteen randomized controlled trials [19, 24–36] compared the efficacy of probiotic-assisted antibiotic therapy for BV with antibiotics alone (or plus placebo), including data from 1662 patients with BV. The cure rate was 72.98% (624/855) in the antibiotics + probiotics group and 62.70% (506/807) in the antibiotics (+placebo) group, with P = 0.009, reaching a statistically significant difference. The results showed that RR was 1.23 with 95% CI (1.05, 1.43). However, the results were heterogeneous (I^2^ = 83%, P < 0.00001), indicating the combined analysis could not be carried out directly, and the subgroup analysis was needed (Fig. 4A).

G2: Three randomized controlled trials [21, 36, 37] were conducted to compare the effects of probiotics and antibiotics. Among them, 92 cases (64.79%) were cured in the probiotics group and 127 cases (77.44%) were cured in the antibiotics group. There was no significant difference in the cure rate of BV between the two treatments (P = 0.72), and there was evidence of obvious heterogeneity (I^2^ = 92%, P < 0.00001), therefore random effect analysis was used for further analysis. In conclusion (RR = 1.12, 95% CI (0.60, 2.07)), the result of G2 analysis cannot be considered that probiotics alone is more effective in treating BV than using antibiotics alone. The number of articles in G2 is so small that we cannot make further analysis. Therefore, more studies are needed to compare the efficacy of antibiotics alone versus probiotics alone in the treatment of BV (Fig. 4B).

G3: Three randomized controlled trials [38–40] compared the efficacy of probiotics with placebo, involving a total of 125 eligible patients with BV. In the probiotics group, 39 out of 70 patients were cured (55.71%), compared to 1 out of 55 patients (0.02%) in the placebo group (P < 0.0001, indicating a statistically significant difference in cure rate). The results of G3 analysis shown I^2^ = 0% and P = 0.68, indicating that there was a low heterogeneity in the included studies, so a fixed model was used to analyze G3, with a result of RR equaled to 15.20 with 95% CI (3.87, 59.64). We can extrapolate from these results that probiotics might has a therapeutic effect on BV compared to placebo (Fig. 4C).

### Subgroup analysis

For studies with large heterogeneity (G1), we set up a subgroup analysis and used a random effects model to explore the causes of heterogeneity (Table 2).

**Table 2:** Summary of subgroup analysis results

Groups	No. trials	No. patients	RR (95 % CI)	P value	I^2^, %	P value of heterogeneity
All studied	14	1662	1.23 (1.05, 1.43)	0.009	83	< 0.00001
Administration route of probiotics						
Oral	5	351	1.63 (1.19, 2.22)	0.002	71	0.008
Vagina	8	1282	1.11 (0.96, 1.28)	0.17	79	< 0.0001
Oral plus Vagina	1	29	0.52 (0.24, 1.16)	0.11	/	/
Diagnosis standards						
Nugent score	7	579	1.01 (0.88, 1.16)	0.89	50	0.06
Amsel’s criteria	4	554	1.81 (0.90, 3.64)	0.09	96	< 0.00001
Amsel and/or Nugent score	3	529	1.26 (0.96, 2.29)	0.19	84	0.002
Recruitment area of participants						
Europe	6	529	1.13 (0.93, 1.38)	0.21	73	0.002
Non-Europe	8	1199	1.20 (0.95, 1.51)	0.13	87	< 0.00001
Follow-up time						
Short-term (≤ 1 month)	11	1149	1.21 (1.01, 1.44)	0.04	84	< 0.00001
Long-term (≥ 1 month)	3	513	1.35 (0.87, 2.10)	0.18	85	0.001
Species of probiotics						
Single species	6	995	1.19 [0.99, 1.43]	0.06	85	< 0.00001
Multiple species	8	667	1.25 [0.93, 1.69]	0.14	83	< 0.00001
Use of *L. rhamnose*						
Yes	11	987	1.28 [1.02, 1.60]	0.03	84	< 0.00001
No	3	675	1.07 [0.92, 1.25]	0.37	64	0.06
Dosage of probiotics						
< 1 × 10^9^	7	929	1.03 [0.94, 1.12]	0.57	42	0.11
≥ 1 × 10^9^	7	733	1.54 [1.13, 2.08]	0.006	77	0.0002

There were no significant differences in other subgroups, such as vaginal administration of probiotics, diagnostic criteria, recruitment areas and species of probiotics. Although the results of short-term follow-up were statistically significant, the removal of any study could not reduce its heterogeneity, and the high heterogeneity made the results unreliable.

Although studies of oral administration to probiotics had great heterogeneity (P = 0.003, I^2^ = 72%), but it was statistically significant (P = 0.0001). Sensitivity analysis would be carried out in the next step to further explore the cause of heterogeneity.

The results of *L. rhamnose* group and high dose group were statistically significant (P = 0.03, P = 0.006), but mainly affected by route of administration. High-dose probiotics (≥ 1 × 10^9^ CFU) was more effective than low-dose probiotics (< 1 × 10^9^ CFU). When *L. rhamnose* was taken orally, the results were statistically significant (P = 0.04), but the heterogeneity was high (I^2^ = 76% P = 0.0008). When *L. rhamnose* was used in the vagina, the results were not statistically significant. It may be because *L. rhamnose* is an intestinal isolate.

### Sensitivity analysis

Following subgroup analysis of G1, the oral administration route showed higher heterogeneity (I^2^ = 71%, P = 0.008). When Zhang Y.2021 was excluded, the whole oral administration group showed no heterogeneity (I^2^ = 0%, P = 0.43). After being analyzed by fixed effects, the results were statistically significant (RR = 1.93, 95% CI (1.59, 2.35), P < 0.00001). This result indicated that when probiotics was added adjunctively in conventional antibiotic therapy for BV treatment, the cure rate was higher than antibiotic therapy alone (or plus placebo) in oral administration. The heterogeneity of Zhang Y.2021 may come from its research method: vaginal administration of metronidazole and oral probiotic, because the research methods of the other four articles were oral antibiotics and oral probiotics.

## Discussion

The main purpose of this study was to systematically evaluate the clinical efficacy of probiotics in the treatment of bacterial vaginosis. The results showed that the cure rate of probiotics combined with antibiotics in the treatment of BV was better than that of antibiotics alone, there was no difference between probiotics and antibiotics alone, and the efficacy of probiotics alone in the treatment of BV was better than that of placebo. For the use of probiotics, oral probiotics was better than vaginal administration in the treatment of BV, oral administration of *L. rhamnose* was more effective than vaginal in the treatment of BV, high-dose probiotics was more effective than low-dose probiotics, and the effective rate was different in short-term follow-up but not in long-term follow-up. These results might provide a reference for future clinical treatment of BV.

According to this study, the cure rate of patients with bacterial vaginosis treated only with probiotics was significantly higher than that of patients treated with placebo, suggesting that probiotics may play a role in the treatment of BV. Compared with the efficacy of probiotics and antibiotics in the treatment of BV, there was no significant difference in the cure rate between the two groups. The clinical cohort study [37] showed 10-day intravaginal injection of probiotics maintained normal vaginal microbiota for longer, compared with 7-day intravaginal injection of metronidazole. Probiotics could effectively and stably restore vaginal microflora and maintain normal vaginal flora for a longer time, which provided a new idea for the treatment of BV. We also need more research on the comparison of the efficacy of probiotics and antibiotics. Our analysis showed that using probiotics as an adjuvant therapy to antibiotics in BV treatment is effective and promising. When probiotic was given orally, it could be considered that the efficacy of probiotics assisted antibiotics in treating BV was better than that of antibiotics alone. In a meta-analysis published in 2017 [41] there was limited evidence to support the fact that metronidazole combined with probiotic supplements was more effective in the treatment of BV than metronidazole alone. However, two other meta-analyses [42, 43] concluded that, despite limited and weak evidence, probiotics showed beneficial effects as a substitute or combination therapy for BV. A meta-analysis published in 2019 [44] showed that probiotics alone were more effective in treating BV in both short and long term, whereas probiotics after antibiotic treatment was only effective in the short term. In short, appropriate sample size and experimental design are needed to further confirm the effectiveness and safety of this treatment strategy.

The preferred route of administration of probiotics has been controversial. On the one hand, an animal experiment [45] had shown that oral administration was more effective than vaginal administration on GV-induced BV. The anti-BV effect of orally intake of a *Lactobacillus rhamnosus* HN001 (L1), *Lactobacillus acidophilus* GLA-14 (L2) and Lactoferrin RCXTM (PM; RECETA ®) may be due to the regulation of immune response through the gastrointestinal tract by these probiotics rather than the completion or killing of GV through the vagina. According to previous studies [46], the vaginal mucus barrier prevented the drug from approaching the folded vaginal epithelium, by which might affect the therapeutic effect of local drugs. On the other hand, a 16SrRNA gene sequencing-based study [25] concluded that oral probiotics were ineffective because probiotics were rarely detected in both vaginal and fecal microbiota. Our analysis showed that when probiotic was given orally, it could be considered that the efficacy of probiotics assisted antibiotics in treating BV was better than that of antibiotics alone. The effectiveness of probiotic products depends on the number of living cells per administration, while the probiotic dose is not clearly defined [47]. Our study showed that the effect of high-dose probiotics in the treatment of BV was better than that of low-dose probiotics in the treatment of BV. The results of this study showed that the effect of *L. rhamnose* was statistically significant only when it was administered orally. *L. rhamnose* is a common *Lactobacillus* isolated from gastrointestinal tract. It has been proved that *L. rhamnose* HN001 could survive under adverse gastrointestinal conditions and adhered to intestinal mucosa [48]. *L. rhamnose* HN001 showed the ability to regulate the composition of intestinal microbiota [49], but had no significant effect on the diversity and richness of intestinal microbiota [49, 50].

At present, the main focus of RCTs on probiotics in the treatment of BV is still effectiveness, so future research should pay more attention to the safety of probiotics and the comparison of the efficacy of antibiotics alone and probiotics alone in the treatment of BV. This study did not limit the types of antibiotics, the heterogeneity between studies was controlled, and the possible causes of heterogeneity in subgroups were found.

## Conclusion

Our meta-analysis found that probiotics may play an active role as an adjuvant treatment to conventional antibiotic therapy for female bacterial vaginosis. However, we need more high-quality, standardized large-sample randomized controlled trials to verify the efficacy of probiotics. In addition, the side effects of probiotics and the selection of high-quality strains may need to be further studied.

### Limitations

There were non-negligible limitations this study. Although we believed that *Lactobacillus* had an effect on the treatment of bacterial vaginosis, in order to include as many literatures as possible, we did not set restrictions on their usage. The RCT follow-up period included in this study was not long, and the long-term effective rate and recurrence rate cannot be observed, so these variables need to be included in the future to evaluate the effectiveness of probiotics in the treatment of BV.

## Data Availability

The datasets employed in the current study can be available from the corresponding author upon the reasonable request.

## Abbreviations

BV: Bacterial vaginosis
RR: Risk ratio
GV: Gardnerella vaginalis
RCTs: Randomized controlled trials
PROSPERO: Prospective Register of Systematic Reviews
M-H: Mantel–Haenszel
CI: Confidence Interval
NSFC: National Natural Science Foundation of China
